# The ANKLE TRIAL (ANKLE treatment after injuries of the ankle ligaments): what is the benefit of external support devices in the functional treatment of acute ankle sprain? : a randomised controlled trial

**DOI:** 10.1186/1471-2474-13-21

**Published:** 2012-02-16

**Authors:** Suzanne Witjes, Femke Gresnigt, Michel PJ van den Bekerom, Jan G Olsman, Niek C van Dijk

**Affiliations:** 1 Department of surgery, Jeroen Bosch Hospital, Henri Dunantstraat 1, 5223 GZ 's-Hertogenbosch, The Netherlands; 2 Department of orthopaedic surgery, Academic Medical Centre, Meibergdreef 9, 1100 DD Amsterdam, The Netherlands; 3 Department of orthopaedic surgery, Slotervaart hospital, Louwesweg 6, 1066 EC Amsterdam, The Netherlands

## Abstract

**Background:**

Acute lateral ankle ligament injuries are very common problems in present health care. Still there is no hard evidence about which treatment strategy is superior. Current evidence supports the view that a functional treatment strategy is preferable, but insufficient data are present to prove the benefit of external support devices in these types of treatment. The hypothesis of our study is that external ankle support devices will not result in better outcome in the treatment of acute ankle sprains, compared to a purely functional treatment strategy. Overall objective is to compare the results of three different strategies of functional treatment for acute ankle sprain, especially to determine the advantages of external support devices in addition to functional treatment strategy, based on balance and coordination exercises.

**Methods/design:**

This study is designed as a randomised controlled multi-centre trial with one-year follow-up. Adult and healthy patients (N = 180) with acute, single sided and first inversion trauma of the lateral ankle ligaments will be included. They will all follow the same schedule of balancing exercises and will be divided into 3 treatment groups, 1. pressure bandage and tape, 2. pressure bandage and brace and 3. no external support. Primary outcome measure is the Karlsson scoring scale; secondary outcomes are FAOS (subscales), number of recurrent ankle injuries, Visual Analogue Scales of pain and satisfaction and adverse events. They will be measured after one week, 6 weeks, 6 months and 1 year.

**Discussion:**

The ANKLE TRIAL is a randomized controlled trial in which a purely functional treated control group, without any external support is investigated. Results of this study could lead to other opinions about usefulness of external support devices in the treatment of acute ankle sprain.

**Trial registration:**

Netherlands Trial Register (NTR): NTR2151

## Background

### Meaning and diagnosis of ankle injuries

Injury to the lateral ligament complex of the ankle is the most common injury in many sports. About one quarter of all injuries across all sports are ankle injuries. In the United States about one sprain per 10.000 people occur per day, in the Netherlands an annual total of 234.000 ankle sprains caused by sports activities are registered [1,2]. Of these, 47% require some form of medical treatment which leads to an estimated annual cost of more than € 80.000.000, only in the Netherlands [3].

Traditionally, the diagnosis of ankle sprain is based on history and delayed physical examination, 5-7 days after initial trauma [4]. The most common injury mechanism is supination and adduction (called inversion) with the foot plantar flexed. Any additional X-rays are used only to exclude other diagnoses, such as a fracture or disturbance of the joint congruency, based on the Ottawa ankle rules [5].

To determine an apparent rupture, a repetition of the physical examination a few days later is more valid [4]. It is hard to distinguish a rupture (grade II/III ankle injury) from a simple sprain (grade I ankle injury) in the first couple of days after injury, because pain, swelling and muscle tension complicate interpretation of the physical examination. In addition, both swelling and pain have a limited positive predictive value for serious injury in the acute phase. The distinction between a grade I or grade II/III injuries is important because of consequences for both treatment and prognosis. It is suggested that a sprain (grade I ankle injury) does not need any treatment because "functional instability" or recurrent sprain is less frequent after ligament sprains than after ligament ruptures [6].

Generally, the prognosis of ankle injuries is good, whatever treatment is followed [7]. Zeegers has showed that at least 80% of patients in all compared treatment groups were free of complaints after one year [8]. However, there is still potential for improvement in 20% of these patients.

### Treatment strategies

In recent decades the treatment of ankle injuries has been discussed frequently. There were various treatments applied in practice, varying from conservative (e.g., immobilization with plaster) to functional (early mobilization with external assistance) to surgical treatment.

However, the natural history of acute ankle sprains is still not fully known, because good quality comparative studies including a no-treatment strategy, functioning as control group, are lacking. In 2000 Pijnenburg et al. conducted a meta-analysis of randomised controlled trials of existing treatment strategies, in which they also putted results after treatment versus minimal treatment against each other [9]. They found that minimal treatment resulted in more residual pain in the long term. Unfortunately they did not clearly define what they meant by minimal treatment, nor they mentioned whether any form of physiotherapy in this group was followed or not. Also because of insufficient comparable data between the included RCT's, it was impossible to include in this analysis outcome measures like recurrent instability, range of motion or appearance of osteoarthritis.

Although there is no conclusive evidence to determine which treatment strategy gives the best result after acute ruptures of the ankle ligaments, the trend nowadays has clearly shifted in favour of functional treatment [10]. Immobilization of the ankle (of at least four weeks) leads to worse results than functional treatment [11]. Moreover, a functional treatment is cheap and simple to apply [12]. That is the main reason there has become more reluctance to surgery, besides the fact that of course an operation entails greater risks for complication. In the most recent Cochrane review of 2002, in which different functional treatment strategies were assessed, Kerkhoffs et al. concluded that most of the available data were of insufficient quality. Until now, it is still impossible to conclude which one amongst the various existing functional treatment methods (with or without external support) is superior [11,13].

In the treatment of ankle injuries the meaning of the phenomenon "functional instability" is important. Functional instability is a subjective complaint of the patient feeling that the ankle is not always stable with complaints of giving way. Based on Freeman's concept, damage or loss of the proprioceptive receptors, which may arise due to loss of strength or propriocepsis disturbances, causes this phenomenon, [14,15].

Several studies have shown that consistency between functional instability and objectively diagnosed mechanical instability doesn't exist [16]. Examination by Akbari et al. confirmed the importance of propriocepsis disorders and loss of muscle strength in the occurrence of residual symptoms, because these patients were found to have impaired balance after acute ankle ligament injuries [17]. A physiotherapy study demonstrated that balance significantly improves after a 4-week exercise program [18].

The ABBA-study showed the value of stability exercises after ankle injury, in which balancing exercises offered benefit in preventing recurrences of ankle ligament injuries among volleyball players [19]. Recently they published results of their follow-up study (2Bfit). In that study 256 athletes (not only volleyball players) followed an eight-week unsupervised propriocepsis training programme. This intervention appeared to result in a reduction of recurrences of 35% compared to a control group [20]. Moreover, Bleakly et al. recently claimed that an accelerated exercising protocol, started during the first week after acute ankle sprain, improved ankle function on short term, without difference in adverse events [21]. Research from the Erasmus Medical Centre Rotterdam, The Netherlands, finally showed that it seems not necessary to perform these exercises under supervision of a physiotherapist, because compared to individually performed exercises, exercises led by physiotherapy did not show any benefit in preventing relapse or subjective recovery within one year after the ankle injury [22].

The advantages of external support resources in the treatment of acute ankle injury are not totally clear. However, several studies showed a possible value in preventing recurrent ankle injuries, especially in high-risk athletes. In 2006 Beynnon described the potential benefit of using braces and bandages, but they never compared their results to a control group without external support [23]. In our opinion the potential advantage of external support devices has to be totally clear before accepting the well-known possible disadvantages of these treatment modalities like skin irritation, dependency and additional treatment costs.

### Objective

The overall aim of the ANKLE TRIAL is to compare results of three different functional treatment options for acute ankle injury. Special focus is on the potential value of external support devices (ankle brace or tape) in addition to a purely functional treatment strategy existing of balance and coordination exercises.

### Hypothesis

Our hypothesis is that treatment with tape or brace, in addition to balance and coordination exercises, will not lead to better result in comparison with purely functional treatment without using external support devices (after minimal 6 months). A difference of more than 10% is regarded as clinically relevant.

### Methods/design

The CONSORT statement is followed to describe the design of this study [24].

### Trial design

**Figure 1:**
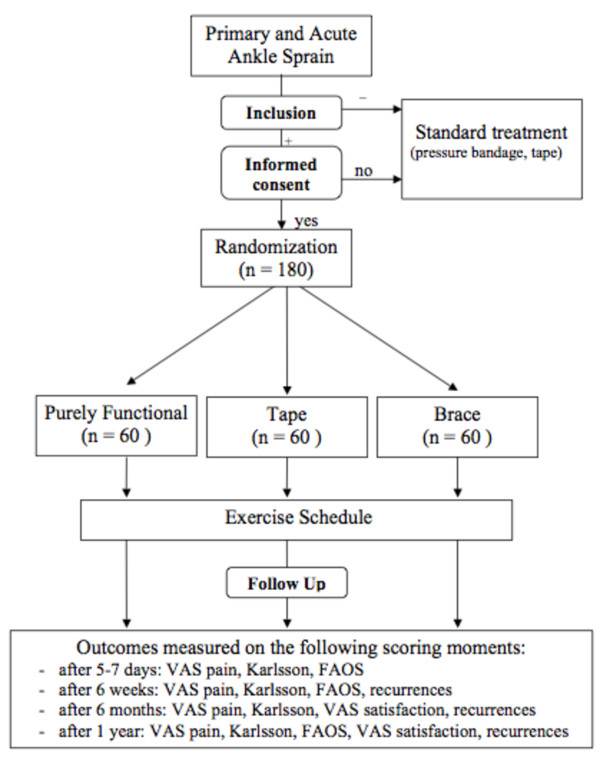
**ANKLE TRIAL flow chart (inclusion, randomization and follow-up)**.

The ANKLE TRIAL is designed as a prospective open randomized multi-centre trial with a one-year follow-up in which we compare three different functional treatment strategies of acute ankle sprains. A residual-free healing and prevention of chronic symptoms and functional instability will be pursued. Therefore, results during follow-up primarily will be evaluated with a statistically validated scoring scale, which measures ankle joint function in the rehabilitation phase after acute ligament injury, according to Karlsson and Peterson [25]. In this scale a maximum of 90 points can be given in following categories: pain (20), swelling (10), instability (subjective) (15), stiffnes (5), stair climbing (10), running (10), work activities (15), and use of support device (5). Selected patients will be randomized in three treatment groups, as showed [Figure 1] with an allocation ratio of 1:1:1. For follow-up patients will return to the hospital after five to seven days (baseline), after six weeks and after one year. After six months patients will be contacted by telephone [Table 1].

**Table 1:** Follow-up moments and outcome measurement

	VAS pain	Karlsson	FAOS	VAS satisfaction	Number of recurrences
< 24 h	+	-	-	-	-

5-7 days	+	+	+	-	-

6 weeks	+	+	+	-	+

6 months	+	+	-	+	+

1 year	+	+	+	+	+

### Participants

Adult and healthy patients with acute (within 24 h), single sided and first inversion trauma of the lateral ankle ligaments will be eligible for inclusion. Patients will be excluded if they do not master the Dutch language, if they are low ambulatory or if there is another (or secondary) diagnosis than lateral ankle sprain (e.g. fracture of the ankle). If necessary, × rays will be performed to exclude ankle fractures, according to the Ottawa ankle rules. All lateral ankle ligament injuries will be included, regardless of severity, as in the acute stage it is hard to distinguish between. After 5-7 days, following the case record form, a delayed physical examination according to Van Dijk [4] will take place and function scores according to De Bie et al. [26] will be determined. From here we can distract the severity of the injuries, which will be important for sub analysis.

### Interventions

The first treatment group will receive pressure bandage and RICE (Rest, Ice, Compression, Elevation) therapy for 5 to 7 days followed by tape treatment for 6 weeks, the second group will be treated by pressure bandage and RICE therapy for 5 to 7 days followed by brace treatment for 6 weeks and the third group RICE therapy and purely functional treatment. The last group will not be given pressure bandage or external support, except for an elastic sock if desired.

In addition, patients in all three groups will receive the same ankle exercise schedule, based on regular existing physiotherapy protocols [Table 2]. Only the first two days after the inversion trauma patients (of all groups) are allowed to take Paracetamol for pain relieve as necessary. After two days they can start weight bearing guided by pain.

**Table 2:** Stepwise exercise schedule with training modality accents per phase

	Training Modalities							
**Step**	**Time**	N*	**Range of motion**	**Weight bearing**	**Walking**	**Stretching**	**Strength**	**Balance**

1	0-2 days	1-3	+++	minimal	-crutches	-	-	-

2	3-10 days	4-6	+	+	+ guided by pain			

3	11-21 days	7-10	++	++	++ incl running	+	+	++ one leg standing

4	3-6 weeks	11-14	++	++	++	+	++	+++

* Number of exercises

### Outcome measures

Primary outcome measure will be the Karlsson scoring scale [25,27]. Secondary outcome measures will be FAOS subscales (pain, other symptoms, Activities of Daily Living (ADL), sports and recreation and quality of life [28]), number of recurrent ankle injuries, Visual Analogue Scale (VAS) of pain and satisfaction, side effects and adverse events.

### Sample size

To prove the hypothesis, the required number of patients was estimated using a sample size analysis, calculated with the contingent primary outcome variable Karlsson scoring scale, according to a study of Boyce et al. [29]. Sample size calculation for this non-inferiority study was based on 3 post hoc comparisons with Bonferroni adjustment. Therefore a one-sided α-level of 0.025/3 = 0.008 was chosen. Limits of equivalence were set at 10 points of the Karlsson score and an expected difference of zero. Based on a standard deviation of 15 and power of 80%, we estimate that 50 patients per group are minimally necessary. With correction of 15% of expected patients to drop out we will include in our study 60 patients per group and 180 patients in total.

### Randomization and blinding

Physicians at the emergency department of both Jeroen Bosch hospital, 's-Hertogenbosch, The Netherlands and Slotervaart Hospital, Amsterdam, The Netherlands will recruit patients. Using the same study protocol for both hospitals, the physician will inform patients, who meet the inclusion criteria, about the study. The selected patients will also receive a brochure with written patient information. To avoid selection bias, only after they have signed informed consent, patients will be allocated to one of the three treatment strategies using a web-based computer randomization tool. Independent and qualified plaster nurses will be responsible for follow-up and data collection of the case record forms after 5-7 days and after 6 weeks. FG will collect data out of the case record forms and will put them into a database (not blinded). SW finally will analyze this objective database.

### Statistical analyses

Baseline characteristics will be presented using descriptive statistics; continuous data will be summarized as mean and standard deviation in case of normal distribution, or as median and range when distribution is skewed. Categorical data will be presented a frequencies and proportions.

The primary analysis will be performed on the main outcome measure of this study, the Karlsson score, at 6 months follow-up. The treatment groups will be compared using an ANOVA test with post hoc pairwise comparisons (with Bonferrini correction). The adjusted confidence intervals from these contrasts will be used to test for non-inferiority using the prespecified non-inferiority margin.

Due to the repeated datastructure, secondary analyses will be performed using linear mixed models on the Karlsson score, FAOS score and VAS scores (change from baseline) to estimate change as a function of time and mean differences between the treatment groups. Akaike Information Criteria (AIC) will be used as an indicator for model fit. The number of recurrent ankle injuries and adverse events will be analyzed using a *X*^2 ^test or log-rank test, when appropriate. Analyses will be based on the intention-to-treat principle and performed in PASW statistics 18 (SPSS Inc. Chicago, IL). Statistical uncertainty will be quantified by 95% confidence intervals.

## Discussion

The ANKLE TRIAL is a randomized controlled trial in which a purely functionally treated (control) group, without any form of external support, will be investigated and compared with two external support treatment (intervention) groups. Recruitment of patients in Jeroen Bosch Hospital has started since January 2010 and in Slotervaart Hospital since July 2011. Recruitment will be finished when 180 patients have been included, which we expect will be at the end of 2011. We expect to publish initial results of our study in 2013.

### Impact of results

The results of this study can lead to a changing view on treatment of ankle sprains. Currently a new guideline concerning treatment of acute ankle sprain has been introduced in The Netherlands, based on latest insights from the literature [29]. The developers of this guideline concluded that rehabilitation of athletes after acute inversion injury of the ankle should consist of varied exercises, which will improve propriocepsis, strength and coordination as well as maintaining function of the extremity.

Furthermore, a brace or tape was recommended in the (sub) acute phase following the diagnosis of acute ankle ligament injury. Also they stated that there is room for tape processing in a (top) sport population, although based on the literature it was not clear which strategy was best. To our knowledge, usefulness of any kind of external support has never been compared to a purely functional treatment without external support [9].

### Registration and ethic approval

This study protocol was registered in the Dutch Trial Register (number NTR2151). It was funded by the COC (Central Education Committee) of the Jeroen Bosch Hospital and supported by Bauerfeind (Haarlem, The Netherlands), who provided the using braces and made publication of this protocol possible. Both did not and will not have any role or influence in the design, conduct, analysis or reporting of this study. The Medical Ethics Committee METOPP in Tilburg, The Netherlands, approved the study design, procedures and informed consent procedure (number NL30075.028.09) and the study will be performed in accordance with the guidelines for Good Clinical Practice.

## Competing interests

The authors declare that they have no competing interests.

## Authors' contributions

JO conceived and designed the study. SW designed the study, worked it out and wrote the manuscript. FG will be responsible for data acquisition and revised the manuscript. MB provided advises on this study and revised the manuscript. CD provided advises on this study. All authors have read and approved the final manuscript.

## Authors' information

JO is trauma surgeon in the Jeroen Bosch Hospital. SW is resident orthopedic surgery, worked at the department of surgery in Jeroen Bosch hospital from 2008 to 2009 and is working in departments of orthopedic surgery since 2010, at first in Slotervaart Hospital and since July 2011 in AMC. FG is resident emergency physician at Jeroen Bosch Hospital. MB is last year resident orthopedic surgery and PhD candidate on ankle ligament injuries in orthopedic training cluster AMC. CD is head of the orthopaedic surgery department in AMC and professor in orthopaedic surgery, focused on ankle pathology.

## Pre-publication history

The pre-publication history for this paper can be accessed here:

http://www.biomedcentral.com/1471-2474/13/21/prepub
